# Ecological Variation in Species Composition and Attachment Preferences of Ixodid Ticks Infesting *Bos taurus* in the Eastern Cape Province, South Africa

**DOI:** 10.3390/microorganisms14051046

**Published:** 2026-05-06

**Authors:** Mpisana Zuko, Nyangiwe Nkululeko, Yawa Mandla, Slayi Mhlangabezi, Jaja Ishmael

**Affiliations:** 1 Department of Livestock and Pasture Science, University of Fort Hare, Private Bag X1314, Alice 5700, South Africa; ijaja@ufh.ac.za; 2 Department of Agriculture and Animal Health, Florida Science Campus, University of South Africa, Johannesburg 2006, South Africa; 3 Department of Agricultural Sciences and Game Management, Nelson Mandela University, South Campus, Gqeberha 6001, South Africa; 4 Döhne Agricultural Development Institute, Private Bag X15, Stutterheim 4930, South Africa; mandlaayawa@gmail.com; 5 Centre for Global Change (CGC), University of Fort Hare, Private Bag x1313, Alice 5700, South Africa; mslayi@ufh.ac.za

**Keywords:** ixodid ticks, cattle, *Bos taurus*, tick ecology, species composition, attachment sites, host–parasite interactions, Eastern Cape, livestock health, vector ecology

## Abstract

Generally, ixodid ticks are important ectoparasites of cattle, including those in smallholder production systems in the Eastern Cape Province, where varying environmental conditions influence their distribution and feeding behaviour. This study investigated ecological variation in tick species composition and attachment site preferences in *Bos taurus* cattle across coastal and inland areas of the Eastern Cape Province, South Africa. Ticks were collected from cattle of different ages, sexes, breeds, and body condition scores. Sampling was conducted prior to acaricide treatment, and ticks were manually removed from standard predilection sites on each animal. Specimens were preserved in 70% ethanol and later identified morphologically at the Döhne Agricultural Development Institute Laboratory. Data were analysed using generalized linear mixed models with a negative binomial distribution to assess the effects of host and environmental factors on tick burden. Descriptive statistics were used to summarise species composition, while inferential statistics were applied to evaluate differences in infestation levels across host-related and spatial variables. A total of 3250 adult ixodid ticks were collected from cattle. The most prevalent species was *Rhipicephalus* (*Boophilus*) *decoloratus* (39.7%), followed by *Rhipicephalus evertsi evertsi* (21.0%), *Amblyomma hebraeum* (17.7%), *Hyalomma rufipes* (5.8%), *Ixodes pilosus* (5.8%), *Rhipicephalus* (*Boophilus*) *microplus* (4.5%), *R. appendiculatus* (3.0%), and *R. simus* (2.5%). Tick burdens were significantly higher in the coastal zone (85 ± 7.5) than in semi-arid inland areas (62 ± 5.9). Attachment site analysis showed significantly higher infestation levels (*p* < 0.05) on the udder/scrotum compared to other body regions. This study provides baseline information on tick species composition and attachment site ecology in cattle, contributing to improved understanding of host–parasite interactions and supporting the development of targeted, region-specific tick control strategies.

## 1. Introduction

Ticks are among the most significant ectoparasites affecting livestock worldwide, causing direct harm through blood-feeding, skin irritation, and reduced productivity, as well as indirect losses through the transmission of tick-borne pathogens. In cattle, tick infestations contribute to declines in weight gain, milk production, hide quality, and overall health, while increasing production costs and economic losses for farmers and the livestock industry. These impacts are particularly severe in sub-Saharan Africa, where ecological conditions and livestock management systems favour high tick burdens and where tick-borne diseases such as babesiosis, anaplasmosis, and theileriosis remain endemic [1,2].

In South Africa, cattle production systems occur across diverse agro-ecological zones, resulting in spatial variation in tick distribution and abundance [3,4,5]. The Eastern Cape Province (ECP), with its contrasting coastal humid zones and semi-arid inland environments, provides a suitable setting for examining how ecological conditions influence tick species composition and attachment site preferences on cattle (*Bos taurus*) [6,7]. Coastal areas are typically characterised by higher humidity, moderate temperatures, and dense vegetation, which favour tick survival and development. In contrast, inland areas experience lower rainfall, seasonal drought, and sparse vegetation, which may limit tick abundance and seasonal activity. Understanding these ecological contrasts is essential for informing targeted and context-specific tick management strategies.

Morphological characteristics of ixodid ticks, including body size, scutum features, mouthpart structure, and sexual dimorphism, are important for species identification and reflect adaptations that influence attachment efficiency and feeding behaviour [8,9]. For instance, long-mouthed ticks such as *Amblyomma hebraeum* can attach more deeply into host skin, while shorter-mouthed species such as *Rhipicephalus* (*Boophilus*) *decoloratus* are highly efficient in mass infestation under favourable environmental conditions. In this study, however, morphology refers strictly to descriptive diagnostic identification traits used for species determination, rather than quantitative morphometric analysis. These traits remain essential for accurate taxonomic classification and ecological interpretation.

Attachment site selection is another key ecological trait influencing tick survival and feeding success. Preferred sites such as the udder, scrotum, ears, perineum, and dewlap are associated with thin skin, high vascularisation, and reduced grooming access, providing optimal conditions for tick attachment and feeding [3,4,5,6,7,8,9,10]. These patterns have direct implications for host–parasite interactions and the epidemiology of tick-borne diseases, as well as for the development of targeted control strategies.

Although ticks are widely recognised as important constraints to livestock productivity, there remains limited integrated information on species composition and attachment site preferences of ixodid ticks infesting *Bos taurus* in South Africa across contrasting agro-ecological zones. In particular, few studies have jointly assessed how ecological variation and host-related factors (age, sex, breed, and body condition) influence tick burden and attachment patterns. Previous studies in the Eastern Cape have documented the presence of key species such as *R.* (*Boophilus*) *decoloratus* and *A. hebraeum*, but have not fully explored their ecological distribution in relation to host–environment interactions and site-specific attachment behaviour. The objective of this study was to assess ecological variation in ixodid tick species composition and attachment site preferences on *Bos taurus* cattle in coastal and inland agro-ecological zones of the Eastern Cape Province, South Africa.

## 2. Materials and Methods

### 2.1. Ethical Clearance

Before data collection, ethical clearance was granted by the Research Ethics Committee of the University of Fort Hare (Reference: 202125552-KT-ZM; 19 June 2024). All procedures complied with the moral standards for animal experimentation outlined by the Society for the Prevention of Cruelty to Animals (SPCA) and the [11] (2013).

### 2.2. Study Sites

**Figure 1:**
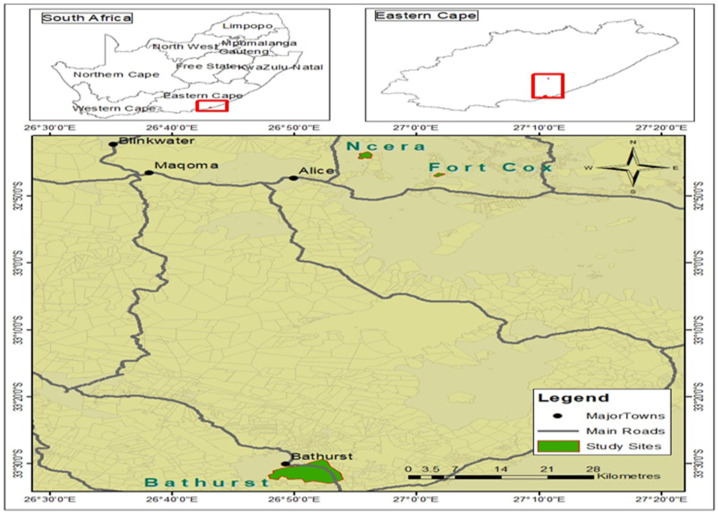
Map of the three agroecological study sites in the Eastern Cape Province.

The study was conducted at three sites in the Eastern Cape Province (ECP) of South Africa (Figure 1), selected to represent contrasting agro-ecological zones. Tick sampling was carried out during the spring and summer seasons (September to February), corresponding to the peak period of tick activity in the region, when higher temperatures and humidity favour tick survival, reproduction, and host infestation. Fort Cox College (32°44′ S, 27°02′ E; altitude: 1430 m), an inland site, receives 500–800 mm of annual rainfall, with temperatures ranging from 4–6 °C (minimum) to 29–32 °C (maximum). During the sampling period (spring–summer), temperatures were generally moderate to warm, providing favourable conditions for tick activity. The vegetation is classified as Buffels Thicket (AT 12), dominated by grasses such as *Digitaria argyrograpta* and *Eragrostis curvula*, and shrubs including *Melolobium burchellii* and *Themeda triandra*. Ncera village, located near Honeydale Research Farm at the University of Fort Hare in Alice (32°8′ E, 26°85′ S; altitude: 500 m), receives an average annual rainfall of approximately 480 mm. The maximum and minimum temperatures range between 18–37 °C and 3–13 °C, respectively. During the sampling period, temperatures were relatively high, particularly in summer, which may enhance tick proliferation. The vegetation is classified as Bhisho Thornveld and is dominated by tree species such as *Maytenus polyacantha*, *Scutia myrtina*, and *Vachellia karroo*. Common grasses include *Cynodon dactylon*, *Digitaria eriantha*, *Eragrostis plana*, *Heteropogon contortus*, *Hyparrhenia hirta*, *Sporobolus africanus*, and *Themeda triandra* [12,13].

Bathurst Research Station (33°30′ S, 26°49′ E; altitude: 708 m), representing a coastal site, receives approximately 624 mm of annual rainfall, with temperatures ranging from 13–29 °C (maximum) and 1–12 °C (minimum). During the spring and summer sampling period, the area experiences mild to warm and relatively humid conditions, which are conducive to tick survival and development. The vegetation is classified as Kowie Thicket (AT 8), characterised by dense succulent shrubs, woody lianas, and evergreen trees on moist slopes. Dominant vegetation includes succulent species such as Euphorbia and Aloe, as well as small trees like *Vachellia karroo*, *Schotia afra*, and *Sideroxylon inerme*, and shrubs such as *Coddia rudis*, *Ehretia rigida*, *Grewia occidentalis*, and *Scutia myrtina* [14,15].

### 2.3. Study Design and Sampling

A cross-sectional survey was conducted during peak tick activity (spring and summer). A total of 205 cattle were randomly sampled from three study sites: 115 animals from inland regions and 90 from the coastal zone. Although this sampling was designed to reflect regional variation, it is important to note that only one herd was sampled per agro-ecological zone. This introduces a key limitation, as observed differences between zones may be confounded by herd-level management practices (e.g., acaricide application frequency, grazing management, and animal handling) and therefore cannot be attributed solely to ecological variation. This limitation reduces the strength of inference regarding true agro-ecological effects and highlights the need for broader multi-herd sampling in future studies to improve external validity. Information on age, breed, and sex was collected during the sampling process. Age was estimated by an experienced livestock handler using dentition patterns and cross-verified with farm records. The animals were relatively homogeneous in age (≥24 months) and live weight (≥350 kg), which helped reduce within-group variability. Body condition scores (BCS) were assessed on a five-point scale [12] and classified as poor (1–2), intermediate (3–4), or good (5) according to [13]. Cattle were grouped by age into two categories: young (<3 years) and older (>3 years), based on farm records.

### 2.4. Tick Collection and Categorization

The study animals were randomly selected and included cattle of different age groups (≤3 years and >3 years) and both sexes. Sampling was conducted prior to dipping to avoid the direct influence of acaricide treatment on tick burden. However, it should be noted that only one herd was sampled per agro-ecological zone; therefore, differences observed between zones may reflect not only environmental variation but also herd-level management practices. Ticks were collected from cattle following the standard method described by [14]. Each animal was examined for 30 min by a trained team comprising experienced animal health technicians and researchers with expertise in tick collection and morphological identification, focusing on seven predilection sites: head, ears, neck, back, abdomen (including udder or scrotum), tail, and legs. Adult ticks were carefully removed using blunt-tipped forceps under proper restraint conditions to minimise discomfort to the animals. In the inland agro-ecological zones, acaricides are routinely applied on a fortnightly basis. However, for this study, the last acaricide treatment was administered three months prior to tick collection. This factor was taken into account during sampling, as it may influence tick infestation levels and species composition.

All collected specimens were preserved in 70% ethanol and transported to the Döhne ADI Laboratory for identification. Ticks were identified morphologically to species level, with adult males and females distinguished using the standard taxonomic keys of [8] (2003). For each animal, tick counts per predilection site and developmental stage were recorded. Tick infestation levels were then analysed using standard parasitological parameters as defined by [15] to enable epidemiological comparison as follows:

▪ Prevalence (%)—the percentage of infested animals;
▪ Mean intensity—the average number of ticks per infested animal;
▪ Mean abundance—the average number of ticks per examined animal (including uninfected animals).

Additionally, infestation levels were categorized according to the classification by [16]: low (1–30 ticks per animal), moderate (31–60 ticks per animal), and high (>61 ticks per animal). This categorization enabled assessment of both regional differences (coastal vs. inland) and host-related variation (breed, age, sex, and body condition), although interpretation of regional comparisons should consider the limitation of single-herd sampling per agro-ecological zone.

#### Justification of Morphological Identification and Future Molecular Validation

Tick identification in this study was conducted using well-established morphological keys [8], which remain the standard approach in field-based epidemiological studies. Diagnostic features such as scutum ornamentation, capitulum structure, mouthpart length, festoons, and anal groove position were carefully examined by trained personnel. Although molecular techniques such as COI gene sequencing provide higher taxonomic resolution, resource limitations prevented their application in the current study. Future research will incorporate molecular approaches to validate species identification and explore genetic diversity among tick populations.

### 2.5. Statistical Analysis

All statistical analyses were performed using Python (version 3.14, 2022) as described by [17] (2022). The analytical approach was aligned with the study objectives, which aimed to (i) compare tick species composition and abundance across agro-ecological zones, (ii) evaluate host-related risk factors, and (iii) assess attachment site preferences.

#### 2.5.1. Descriptive Analysis (Objective 1: Species Composition and Distribution)

Tick species composition and abundance were summarised using descriptive statistics, including frequencies, percentages, and proportions. This was used to describe overall species distribution across the study population and between agro-ecological zones.

#### 2.5.2. Generalized Linear Mixed Model (GLMM) for Tick Burden Analysis (Objectives 2 and 3)

Tick burden (count data) was analysed using a Generalized Linear Mixed Model (GLMM) with a negative binomial distribution and a log link function to account for overdispersion.

The model structure was specified as:

$$y_{ijk}∼\mathrm{NegBin}(\mu_{ijk},\theta )$$

where $y_{ijk}$ represents the tick count for the i-th animal in the j-th region and k-th host category.

The linear predictor was defined as:

$$log(\mu_{ijk})=\beta_{0}+\beta_{1}\mathrm{Age}+\beta_{2}\mathrm{Sex}+\beta_{3}\mathrm{Breed}+\beta_{4}\mathrm{BCS}+\beta_{5}\mathrm{Region}+u_{i}$$

where:

▪ Age, sex, breed, body condition scoring (BCS), and region were included as fixed effects;
▪ $u_{i}∼N(0,\sigma^{2})$ represents the random effect for individual animal, accounting for repeated tick counts across predilection sites and within-animal clustering.

Least squares means (LSMEANS) were used for pairwise comparisons among categorical variables, and statistical significance was assessed using adjusted comparisons. BCS values were square-root transformed prior to analysis to improve normality and model fit. A one-way ANOVA was conducted as an exploratory analysis to compare mean tick burdens between agro-ecological zones where assumptions of normality and homoscedasticity were satisfied. This analysis was used for descriptive comparison rather than primary inference. Binary logistic regression was used to assess associations between tick infestation status (infested = 1, non-infested = 0) and explanatory variables including region, age, sex, breed, and BCS. Results were expressed as odds ratios (OR) with 95% confidence intervals (CI). All statistical tests were two-tailed, and statistical significance was set at *p* < 0.05.

## 3. Results

The results indicate the presence of a diverse assemblage of tick species infesting cattle (Table 1), with a distinct morphological characteristic that facilitates species identification. The majority of the identified ticks belong to the genus *Rhipicephalus*, including the subgenera *Boophilus*, *Amblyomma*, and *Hyalomma*. The species under these genera generally exhibit brown to dark coloration. *Amblyomma hebraeum* and *Hyalomma rufipes* are distinguished by their larger body size and longirostrate (long mouthparts). *Amblyomma hebraeum* displays an ornate scutum with bright yellow-orange enamel-like markings, while *Hyalomma rufipes* is characterized by a narrow body and banded legs. *Ixodes pilosus* differs from the other species by lacking festoons and eyes and possessing an anterior anal groove, which is characteristic of the genus Ixodes. These features distinguish it clearly from the other genera identified in the study.

A total of 3250 ixodid ticks were collected from cattle, comprising eight species (Table 2). *Rhipicephalus* (*Boophilus*) *decoloratus* was the most common species, making up 39.7% of all ticks, followed by *R. evertsi evertsi* (21.0%) and *Amblyomma hebraeum* (17.7%). These three species accounted for most of the tick population. The other species were less common. *Hyalomma rufipes* and *I. pilosus* each contributed 5.8%, while *R. microplus* accounted for 4.5%. The least common species were *R. appendiculatus* (3.0%) and *R. simus* (2.5%) (Table 2).

Table 3 presents the distribution and preferred attachment sites of ticks collected from *Bos taurus* cattle in the coastal and inland regions of the ECP, South Africa. The udder (♀) and scrotum (♂) were the most heavily infested predilection sites, accounting for 41.8% of the total ticks collected. The mean tick burden at this site was significantly higher in the coastal zone (85 ± 7.4) compared to the inland zone (62 ± 5.9) (*p* < 0.05). This site was classified as having a high infestation level. The perineal region represented 13.6% of the total ticks and showed mean burdens of (47 ± 4.2) in the coastal zone and (31 ± 3.8) in the inland zone. The difference between zones was statistically significant (*p* < 0.05), and infestation at this site was categorized as high to moderate. Ticks collected from the ears accounted for 12.4% of the total, with mean burdens of 36 ± 3.6 (coastal) and 22 ± 2.9 (inland). The neck contributed 11.3% of ticks, with means of 32 ± 3.1 in the coastal zone and (19 ± 2.7) inland. Similarly, the dewlap represented 9.7% of total ticks, with 28 ± 2.8 (coastal) and (17 ± 2.3) (inland). These predilection sites showed moderate infestation levels, and the differences between regions were significant (*p* < 0.05).

Lower infestation levels were observed on the dorsal surface and lateral body surfaces. The dorsal surface accounted for 6.4% of total ticks, with mean burdens of (18 ± 2.2) in the coastal zone and (11 ± 1.7) inland. The lateral body surfaces represented 4.8% of ticks, with mean values of (14 ± 1.9) (coastal) and (9 ± 1.4) (inland). These sites were classified as having low infestation levels. Across all predilection sites, tick burdens were consistently higher in the coastal zone than in the inland zone and means within columns bearing different superscripts differed significantly at *p* < 0.05.

Table 4 shows that across all host categories, cattle from the coastal region had markedly higher tick burdens than those from the inland region. For example, younger cattle (<3 years) recorded 62 ± 5 ticks in the coastal region compared to 28 ± 3 inland (*p* = 0.05), while older cattle (>3 years) had 88 ± 7 ticks in the coastal region versus 45 ± 4 inland (*p* = 0.01). Older cattle (>3 years) carried higher mean tick loads than younger cattle (<3 years) in both regions. In the coastal region, older animals had 88 ± 7 ticks compared to 62 ± 5 in younger animals. Similarly, inland older cattle recorded 45 ± 4 ticks compared to 28 ± 3 in younger cattle. Females exhibited higher tick burdens than males in both regions. In the coastal region, females had a mean burden of 80 ± 6 compared to 65 ± 6 in males (*p* = 0.05). Inland, females recorded 44 ± 4 ticks compared to 32 ± 3 in males (*p* = 0.01). This suggests that female cattle are more heavily infested than males. Breed differences were statistically significant. In the coastal region, Nguni cattle had the highest mean tick burden (90 ± 8), followed by crossbreds (84 ± 7) and Bonsmara (76 ± 7). Inland, Nguni also showed the highest burden (47 ± 5), followed by crossbreds (42 ± 4) and Bonsmara (39 ± 4). These differences were statistically significant (*p* ≤ 0.05), indicating that breed has a significant influence on infestation levels. Body condition score showed the strongest association with tick burden. In the coastal region, cattle with poor body condition had the highest burden (95 ± 9), followed by medium (74 ± 6) and good condition animals (58 ± 5) (*p* = 0.001). Similarly, inland cattle with poor condition recorded (52 ± 6) ticks, compared to (37 ± 4) in medium and (29 ± 3) in good condition animals.

## 4. Discussion

The study offers valuable insights into the species composition, abundance, and preferred attachment sites of ixodid ticks infesting cattle (*Bos taurus* Linnaeus, 1758) in the coastal and inland regions of the Eastern Cape Province, South Africa. Eight tick species were identified, highlighting considerable tick diversity. Among these, *Rhipicephalus* (*Boophilus*) *decoloratus* (Koch, 1844) was the most common species, with higher burdens observed in cattle from coastal areas compared to inland sites. The dominance of *R.* (*Boophilus*) *decoloratus* in the coastal region is likely associated with favourable climatic conditions such as higher humidity [18,19], warmer temperatures [20,21], and abundant vegetation that support tick survival and reproduction [22]. However, because sampling was conducted from only one herd per agro-ecological zone, these differences should be interpreted cautiously, as they may reflect not only environmental variation but also herd-level management practices.

The morphological characteristics of ixodid ticks observed in this study are consistent with established diagnostic criteria used for accurate species identification, including features such as the shape of the basis capituli, scutal ornamentation, mouthpart length, and the position of the anal groove [8,18]. Species within the genus Rhipicephalus, particularly *R.* (*Boophilus*) *decoloratus* and *R.* (*Boophilus*) *microplus*, exhibited compact bodies and short mouthparts, aligning with previous reports from southern Africa [19,20]. Similarly, the identification of *Amblyomma hebraeum* based on its ornate scutum and elongated mouthparts, and *Hyalomma rufipes* based on its distinct morphology and banded legs, is consistent with earlier studies highlighting the importance of morphological traits for species differentiation and ecological adaptation [8,21,22]. The distinct morphology of *Ixodes pilosus*, including the absence of eyes and festoons and the presence of an anterior anal groove, further supports its ecological association with humid environments [21,23]. In addition, *R. appendiculatus* and *R. simus* displayed morphological features consistent with their known roles in disease transmission and their association with livestock–wildlife interfaces [19,24]. A limitation of this study is the reliance on morphological identification without molecular confirmation. Although standard taxonomic keys were used, future studies should incorporate molecular tools such as DNA barcoding to enhance species resolution and reproducibility.

The study also identified clear patterns in tick attachment site preferences on *Bos taurus*, with the udder, scrotum, and perineal regions showing the highest infestation levels. These body regions are characterized by thin skin, high vascularization, and reduced accessibility to grooming, providing favourable conditions for tick attachment and feeding [7]. Moderate infestations were observed on the ears, neck, and dewlap, while lower tick burdens were recorded on dorsal and lateral surfaces, likely due to thicker skin, greater exposure, and easier access for grooming. These findings are consistent with previous studies in Africa and Asia reporting similar attachment patterns among ixodid ticks, particularly *Rhipicephalus* species [10,25,26]. Higher tick loads observed in coastal cattle compared to inland cattle suggest that environmental conditions such as humidity, vegetation density, and temperature may influence tick survival and development. Coastal areas generally provide more favourable microclimates for tick proliferation, whereas inland semi-arid areas are less suitable. However, given that only one herd was sampled per agro-ecological zone, these differences cannot be attributed solely to ecological variation, as farm-level management practices (including acaricide application frequency, grazing intensity, and animal handling practices) may have also contributed to the observed patterns. Notably, in this study, coastal cattle were dipped at longer intervals compared to inland cattle, which may have further influenced tick burden differences. This highlights the combined and potentially confounded effects of environment and management on tick distribution.

Age-related differences were evident, with older cattle (>3 years) carrying higher tick burdens than younger animals (<3 years) in both regions. This pattern likely reflects cumulative exposure over time and possible age-related declines in immune responsiveness (immunosenescence), which may increase susceptibility to ectoparasites. Similar findings have been reported in other regions, where older cattle consistently show higher tick infestations [24,27]. Sex-related differences were also observed, with females carrying higher tick loads than males across both ecological zones. This trend may be associated with hormonal influences and physiological stresses linked to reproduction, including pregnancy and lactation, which can modulate immune function and increase susceptibility to parasitism [10,25].

Breed differences were also evident, with Nguni cattle in coastal areas showing higher tick burdens than those in inland regions. Although indigenous breeds such as Nguni are generally considered more tick-resistant due to their adaptive traits, the elevated burdens observed in coastal cattle suggest that environmental conditions and/or management practices may override inherent breed resistance. However, this interpretation should be made cautiously, given the study design limitation of single-herd sampling per zone, which restricts the ability to separate breed effects from farm-specific influences. Similar variability in breed-associated resistance has been reported in other African studies [27,28,29]. Body condition score showed a strong association with tick burden, with poorly conditioned animals carrying significantly more ticks than well-conditioned animals in both zones. This relationship is likely bidirectional: poor body condition may increase susceptibility due to weakened immunity, while heavy tick infestation contributes to weight loss, anaemia, and reduced productivity through blood loss and pathogen transmission [30]. Similar negative associations between body condition and ectoparasite load have been reported in other African livestock systems [5,30].

## 5. Conclusions

This study provides important insights into the diversity, abundance, and distribution patterns of ixodid ticks infesting *Bos taurus* cattle in the Eastern Cape, South Africa. Eight tick species were identified, with *R.* (*Boophilus*) *decoloratus* being the most prevalent. While differences in tick distribution were observed across sampling sites, these patterns should be interpreted with caution, as sampling was conducted from a single herd within each agro-ecological zone. Consequently, the observed variation may reflect not only environmental conditions such as humidity and temperature, but also herd-level management practices. Variations in infestation levels across age, sex, breed, and body condition indicate complex host–environment interactions, with higher burdens observed in older, female, and poorly conditioned animals. The tick’s predilection for warm, well-vascularized body regions further emphasizes patterns of host utilization. Practically, these findings support the implementation of targeted and integrated tick control strategies, including focused control measures on high-risk body regions and vulnerable animal groups. Regular monitoring of tick populations, strategic timing of treatments, and improved farm management practices are essential to enhance control effectiveness. Tailoring interventions to specific agro-ecological zones may further optimize outcomes; however, such recommendations should be considered in light of the study’s limitation of one herd per zone. Furthermore, the reliance on morphological identification without molecular confirmation highlights the need for future studies to incorporate molecular tools such as DNA barcoding to improve species resolution, strengthen diagnostic accuracy, and enhance the reproducibility of findings.

## Figures and Tables

**Table 1:** Key morphological features of ixodid tick species used to identify ticks collected from cattle in the Eastern Cape Province, South Africa.

Tick Species	Body Size	Scutum	Mouthparts	Basis Capituli	Eyes	Festoons	Anal Groove	Legs	Colour
*Rhipicephalus* (*Boophilus*) *decoloratus*	Small	Inornate	Short	Hexagonal	Absent	Present	Posterior	Normal	Dark brown to black
*Rhipicephalus microplus*	Small–medium	Inornate	Short	Hexagonal	Absent	Present	Posterior	Normal	Reddish-brown to brown
*Amblyomma hebraeum*	Large	Ornate, bright enamel-like markings	Very long	Hexagonal	Present	Present	Posterior	Normal	Dark brown to black, scutum with yellow-orange markings
*Rhipicephalus evertsi evertsi*	Medium	Inornate	Short	Hexagonal	Present	Present	Posterior	Banded	Reddish-brown to dark brown
*Hyalomma rufipes*	Large, narrow	Inornate	Long	Hexagonal	Present	Present	Posterior	Banded	Light brown to dark brown
*Ixodes pilosus*	Small	Inornate	Long	Hexagonal	Absent	Absent	Anterior	Normal	Reddish-brown
*Rhipicephalus appendiculatus*	Large	Inornate with subtle ornamentation	Long	Hexagonal	Present	Present	Posterior	Normal	Brown to dark brown
*Rhipicephalus simus*	Large, robust	Inornate	Short	Hexagonal	Present	Present	Posterior	Normal	Dark brown

**Table 2:** Frequency and percentage distribution of tick species identified from cattle in the Eastern Cape Province, South Africa (*n* = 3250).

Tick Species	Frequency (*n*)	Percentage (%)
*Rhipicephalus* (*Boophilus*) *decoloratus*	1291	39.7
*Rhipicephalus* (*Boophilus*) *microplus*	148	4.5
*Amblyomma hebraeum*	577	17.7
*Rhipicephalus evertsi evertsi*	681	21.0
*Hyalomma rufipes*	188	5.8
*Ixodes pilosus*	188	5.8
*Rhipicephalus appendiculatus*	98	3.0
*Rhipicephalus simus*	79	2.5
**Total**	**3250**	**100**

**Table 3:** Preferred locations of ticks collected from *Bos taurus* in coastal and inland regions of the Eastern Cape, South Africa.

Predilection Site	Total Ticks (%)	Coastal Zone (Mean ± SE)	Inland Zone (Mean ± SE)	Infestation Level
Udder (♀)/Scrotum (♂)	41.80%	85 ^e^ ± 7.4	62 ^d^ ± 5.9	Very High
Perineal region	13.60%	47 ^cd^± 4.2	31 ^c^ ± 3.8	High
Ears	12.40%	36 ^c^ ± 3.6	22 ^b^ ± 2.9	Moderate
Neck	11.30%	32 ^c^ ± 3.1	19 ^ab^ ± 2.7	Moderate
Dewlap	9.70%	28 ^b^ ± 2.8	17 ^ab^ ± 2.3	Moderate
Dorsal surface	6.40%	18 ^ab^ ± 2.2	11 ^a^ ± 1.7	Low
Lateral body surfaces	4.80%	14 ^a^ ± 1.9	9 ^a^ ± 1.4	Low

Means in the same column with different superscripts (^abcde^) differ significantly at *p* < 0.05. ♀: Female, ♂: Male.

**Table 4:** Influence of host factors on mean tick burden (Mean ± SE) in *Bos taurus* cattle from coastal and inland regions of the Eastern Cape, South Africa.

Host Factor	Category	Coastal (Mean ± SE)	Inland (Mean ± SE)	*p*-Value
Age	<3 years	62 ^d^ ± 5	28 ^a^ ± 3	0.05 *
	>3 years	88 ^e^ ± 7	45 ^c^ ± 4	0.01 ***
Sex	Male	65 ^d^ ± 6	32 ^ab^ ± 3	0.05 *
	Female	80 ^e^± 6	44 ^c^ ± 4	0.01 **
Breed	Nguni	90 ^e^ ± 8	47 ^c^ ± 5	0.01 **
	Bonsmara	76 ^de^ ± 7	39 ^b^ ± 4	0.05 *
	Crossbred	84 ^e^ ± 7	42 ^bc^ ± 4	0.01 **
Body Condition	Poor	95 ^e^ ± 9	52 ^cd^ ± 6	0.001 ***
	Medium	74 ^de^ ± 6	37 ^b^ ± 4	0.01 **
	Good	58 ^cd^ ± 5	29 ^a^ ± 3	0.05 *

Means in the same column with different superscripts (^abcde^) differ significantly at *p* ≤ 0.05. * *p* < 0.05, ** *p* < 0.01, *** *p* < 0.001

## Data Availability

Data are available upon request from the corresponding authors.
